# Supplementary material for: Microbial community dynamics during historic drought and flood in Great Salt Lake
Source: Appl Environ Microbiol. 2026 Jul 20;92(8):e00234-26. doi: 10.1128/aem.00234-26 (PMC13488349; doi:10.1128/aem.00234-26)
Supplement: Supplementary file 1 [file aem.00234-26-s0001.pdf]

Supplementary Information for Microbial community dynamics during historic drought and flood in the Great Salt Lake

Contents of this file

Text S1: Eddy-resolved 2D Hydrodynamic Model of the Flow through the Breach as the GSL

Legends for other supplementary figures and tables referenced in the main text:

Supplementary Figure S1: Environmental parameters at GSL. (A) Snowfall records from the US Dept. of Agriculture showing snowfall in the year in question. Snowpack peaked at 30 inches / 72 cm snow water equivalent, up from the median peak of ~16 inches / 40.6 cm. (B) Water temperature at sampling sites during the 2022-2023 sampling year. (C) pH at sampling sites during the sampling year. (D) Correlation plot for relationship between environmental parameters. Dot color corresponds to the extent of correlation according to the color scale bar at right. Dot size represents the absolute value of the correlation coefficient. Blank correlations did not meet the correlation significance threshold of $p < 0.05$.

Supplementary Figure S2: Beta diversity plots with constrained principal components of site, season, and origin, then broken down by arm.

Supplementary Table S1: Location ("sampling" subtable) and environmental metadata ("metadata" subtable) across all sites and samples taken for this study. Full 16S amplicon dataset is included with subtables: (a) taxonomoic composition ("taxtab"); (b) sequence abundances by site and season ("seqtab"); (c) raw sequences ("refseqs").

Supplementary Table S2: PERMANOVA results for Origin, site, and season and their effect on beta diversity metrics. Tests were run with vegan::adonis2 in R with 999 permutations.

Supplementary Table S3: Top 200 most abundant taxa with relative abundance statistics. Contains data used to make supplementary Table S2.

Supplementary Table S4: ANCOM-BC statistical output.

Supplementary Table S5: Environmental parameters across the halocline depth profile at the breach using a Niskin bottle (USGS).

Supplementary Table S6: Barcode sequences used for demultiplexing 16S amplicon sequences.

Supplementary Movie S1: Model simulation results for the case with no berm at the Great Salt Lake Breach site (prior to sampling year).

Supplementary Movie S2: Model simulation results for the case with a low berm at the Breach site (fall, winter, and summer of the sampling year).

Supplementary Movie S3: Model simulation results for the case with a high berm (spring of the sampling year).

Text S1: Eddy-resolved 2D Hydrodynamic Model of the Flow through the Breach as the GSL

S1.1: Introduction

Asymmetric mixing in the water column has been observed North of the berm at the GSL breach. We have developed a scaled 2D eddy-resolved computational fluid dynamics model to explain the physical basis of the observation. Previously, Reynolds Averaged Navier-Stokes (RANS) based 3D model of the flow through the breach had been developed to quantify the dynamics of the buoyancy-driven exchange flow [1]. The 3D model captures the large-scale dynamics of the flow, but as the model is based on solving the time-averaged RANS equations, it is unable to resolve the Kelvin-Helmholtz (KH) instabilities at the interface of the exchange flow. Thus, for this study we will solve the incompressible Navier-Stokes equation with the appropriate boundary conditions, resolving all the scales of turbulence and eddies. Solving the NS equations directly without any approximation is also referred as direct numerical simulation (DNS). DNS is computationally costly at high Reynolds number (Re), with cost of simulation being directly proportional to ~ Re^3 [2]. This makes it impossible to conduct DNS at the field-scale. Though, using principles of dynamic similarity we know that if the simulations (or experiments) we conduct are in the same regime as the field, we should expect to see qualitative similarity in the general dynamics of the flow. Thus, all the DNS conducted for the current study will be for a scaled down domain and reduced Reynolds number, allowing us to qualitatively capture the asymmetric mixing observed in the field. To further reduce computational cost, the simulations has been conducted in 2D instead of 3D. This allows us to reduce the computational cost significantly. Long-term evolution of KH instabilities at the interface of two stratified fluids are different between 2D and 3D, especially for turbulence induced mixing [3]. Though, large-scale structures in the flow have been found to evolve over similar time scale in 2D and 3D, showing that 2D simulations are useful for illustrating the growth of instabilities at the interface [4]. Thus, for the current study we have used 2D DNS of buoyancy-driven exchange flows.

S1.2 Governing Equations

The general form of the mass conservation equation is

$$\frac{1}{\rho}\frac{D\rho}{Dt} + \vec{\nabla} \cdot \vec{u} = 0$$

Where $\rho = \rho(x, y, t)$is the fluid density (kg / m^3) $where\ x\ is\ the\ horizontal\ direction\ and\ y\ is\ the\ vertical\ direction,$ $\frac{D}{Dt}$ is the material derivative, and $\vec{u} = \vec{u}(x, y, t)$ is the velocity vector (m/ s). For the incompressible flows under the Boussinesq approximation, the equation reduces to:$\vec{\nabla} \cdot \vec{u} = 0$

(1.1)

Under Boussinesq approximation, the magnitude of the $\frac{1}{\rho}\frac{D\rho}{Dt}$ is small in comparison to the magnitude of the velocity gradients in $\vec{\nabla} \cdot \vec{u}$. Therefore, we can neglect the density variation. The Momentun Conservation equation for the flow is

$$\frac{\partial \vec{u}}{\partial t} + \vec{u} \cdot \nabla \vec{u} = -\frac{\vec{\nabla} \mathrm{P}}{\rho_0} + \frac{\rho - \rho_0}{\rho_0}\vec{g} + \nu \nabla^2 \vec{u} \qquad (1.2)$$

Where $\vec{u}$ is the velocity $(m\ /s\ )$, t is time (s), P = P(x,y,t) is pressure(Pa), ρ is the fluid density(kg/ m^3), ρ_0 the reference density(kg/ m^3), $\vec{g}$ is gravitational acceleration $(m\ /s^2)$ which only acts in the vertical direction, and ν is kinematic viscosity(m^2/s).

For the current study we will consider $\rho_0 = \rho_S$, that is the density of the South-arm (ρ_S) will be used as the reference density for the CFD model. The transport of salt is modeled using the advection-diffusion, and is defined as:

$$\frac{\partial S}{\partial t} + \vec{u} \cdot \nabla S = k\, \nabla^2 S \tag{1.3}$$

Where S = S(x,y,t) is salinity or salt concertation, t is time, $\vec{u}$ is the velocity, k is the molecular diffusivity of salt, and ∇^2 is Laplacian operator. The density field is related to salinity by the linear equation of state

$$\rho = \rho_0(1 + \beta \Delta S) \tag{1.4}$$

or equivalently

$$\rho - \rho_S = \beta(S - S_S) \tag{1.5}$$

Where ρ is the fluid density, ρ_0 is the reference density, S is salinity, and S_S is the salinity of south arm of GSL, and β is the constant expansion coefficient. These are standard set of equations solved for conducting DNS of buoyancy driven flows [5].

S1.3 Non-dimensional Governing Equations

Let us assume that the characteristic length, velocity and density scales are denoted by L_C, U_C, and ρ_c respectively. Accordingly, all velocities, lengths, time, and mass can be non-dimensionalized as follows, where all variables with (~) represent their non-dimensional form.

Parameter	Length	Velocity	Time	Mass
Nondimensionalization	$\tilde{l} = l/L_c$	$\tilde{u} = u/U_c$	$\tilde{t} = t/T_c$	$\tilde{m} = m/(\rho_c L_c^3)$

TableS1.1: Definition of Nondimensional Variables

We assume the mean inflow velocity U_{in} as the characteristic velocity (U_c), total depth of the flow H as the characteristic length (L_c), and the reference density of the exchange-flow system $\rho_0 = \rho_S$, is assumed to be the characteristic density (ρ_c). We non-dimensionalize the governing equations (1.1 to 1.3)

Non-dimensional form of the Mass conservation is defined as

$$\tilde{\vec{\nabla}} \cdot \tilde{\vec{u}} = 0 \tag{1.6}$$

The non-dimensional form of the momentum conservation equation is:

$$\frac{\partial \tilde{\vec{u}}}{\partial \tilde{t}} + \tilde{\vec{u}} \cdot \tilde{\vec{\nabla}} \tilde{\vec{u}} = -\tilde{\vec{\nabla}} p + \frac{1}{Fr_d^2} \tilde{S} \hat{y} + \frac{1}{Re} \nabla^2 \tilde{\vec{u}} \tag{1.7}$$

The non-dimensional quantity of salinity advection diffusion is known as:

$$\frac{\partial \tilde{S}}{\partial \tilde{t}} + \tilde{\vec{u}} \,.\, \tilde{\vec{\nabla}} \tilde{S} = \frac{1}{Re.Sc} \nabla^2 \tilde{S} \tag{1.8}$$

where Reynolds number of the flow (Re) is defined as $\frac{\nu}{U_{in}\mathrm{H}} = \frac{1}{Re}$. Reynolds number of the flow essentially tells if the flow is turbulent or laminar. As the flow at the breach is turbulent, Re will be assumed high enough for the flow in the simulation is turbulent. For the current study, Re = 10,000 has been used. Sc is the Schmidt number of the flow, which parametrizes the relative diffusion of momentum of the fluid to the molecular diffusion the solute (in this case salt) in the fluid. For this current study, Sc is assumed to be 1. *ReSc* can be written is Peclet number (Pe). So the flow at the GSL and the idealized simulation has $Pe >> O(1)$, which makes the regime of the flow being dominated by advective-mixing instead of molecular diffusion. This can also be interpreted as the transverse diffusion timescale H^2/D is substantially smaller than the transverse advective mixing timescale H/U_{in}, where D is the diffusion coefficient of salt. For turbulent flows, this is an appropriate value to use for DNS [5]. The linear equation of state (1.4-1.5) can be rewritten as

$$\frac{\rho-\rho_S}{\rho_N-\rho_S} = \frac{S-S_S}{S_N-S_S} \quad (1.9)$$

Where ρ_N is the density of the North arm of GSL and S_N is salinity of the North arm of the flow. The non-dimensional acceleration due to the gravity can be written as $\tilde{g} = \frac{g\mathrm{H}}{U_{in}^2}$. For the current system, densimetric Froude number is defined as $Fr_d = \frac{U_{in}}{\sqrt{g\left[\frac{\rho_N-\rho_S}{\rho_S}\right]H}}$. Thus, the body force term in equation (1.2) gets non-dimensionalized to the form seen in equation (1.7) though:

$$\left(\frac{\rho-\rho_S}{\rho_S}\right)\frac{g\mathrm{H}}{U_{in}^2} = \frac{1}{Fr_d^2}\left(\frac{\rho-\rho_S}{\rho_N-\rho_S}\right) = \frac{1}{Fr_d^2}\left(\frac{S-S_S}{S_N-S_S}\right) = \frac{1}{Fr_d^2}\tilde{S} \quad (1.10)$$

where $\tilde{S}$ is a non-dimensional scaling of Salinity given as $\tilde{S} = \frac{S-S_S}{S_N-S_S}$.

S 1.4 Scaling of relevant parameters

In this section we will discuss the scaling analysis that resulted in the non-dimensional parameters we used for conducting the simulations for the current study. A schematic of the computational domain has been illustrated in Fig. S1.1.

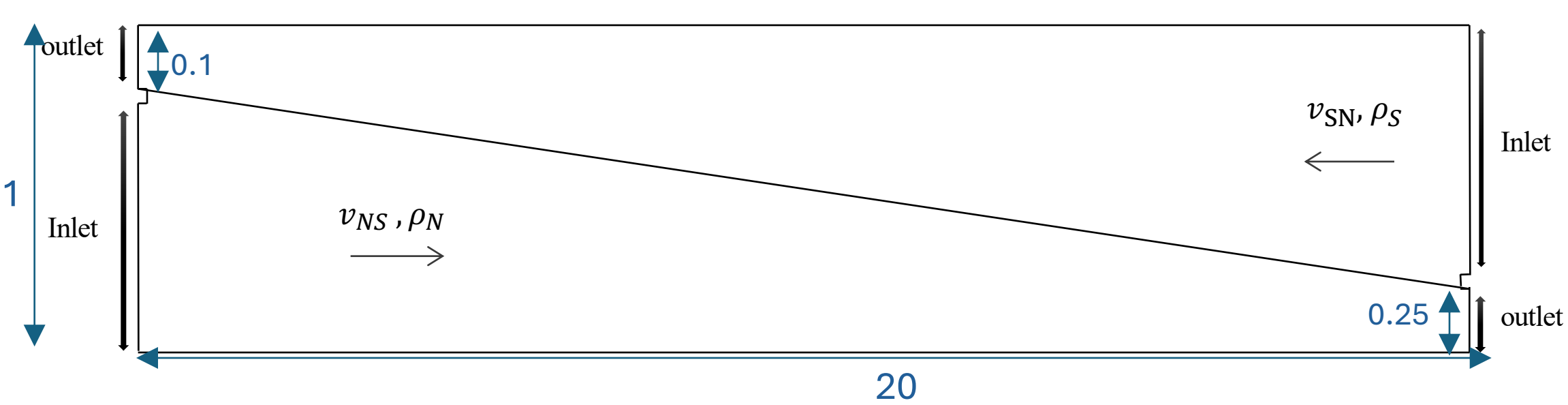

Figure S1.1: Domain for 2D simulation of the buoyancy-driven exchange flow at the breach

The height of the domain is assumed to be equal to that of the depth of the flow at the breach of the GSL, which is around 5 m [1]. The length of the domain is assumed to be around 100 m, which approximately the width of the length of the breach. For more details, please refer to Figure 5 of Rasmussen et al. [1]. Based on the observed and simulated dynamics of the buoyancy-driven exchange flow through the breach (see figures 12-14 in Rasmussen et al.), the inflow-outflow heights of the denser North-South and less dense South-North flows were assumed to be (0.9H and 0.25H) and (0.75H and 0.1H), respectively. The berm at

the breach is on the Northern side of the causeway bridge, and in the computational domain they are place approximately at the correct location (see Fig. S1.2). The average density difference between the southern and northern sides is 84.47 kgm^{-3}, with the South arm density being 1097.75 kgm^{-3}. Based on the fact that the maximum observed velocity of the flow is about 1 m/s and total depth of the flow, the densimetric Fr number of flow is calculated to be $\frac{1m/s}{\sqrt{9.81(m/s^2)*\frac{82.47}{1097.75}*5m}}$ = 0.506. For purpose of this study, as we have maintained realistic buoyancy effects but scaled down the inertial effect, which means reduce the Reynolds number (Re) of the flow. This is a valid assumption to make because in the physics we are trying to resolve, buoyancy impedes mixing across the interface whereas inertial effects generated instabilities at the interface that can lead to mixing of the denser North arm water into the less dense South arm flow. If we can qualitatively show that this can occur at relatively low Re, we can expect similar asymmetric mixing to occur at field-scale Re (~ 10^6). While lowering the Re of the simulated flow, the final parameter that is pertinent to this scaled down system is the ratio of the momentum of the South-North and Nort-South flow. It is important to maintain the momentum ratio (MR) observed in the field, as it is this ratio that governs the relative

Momentum Ratio

$$\text{Momentum Ratio} = MR = \frac{M_{SN}}{M_{NS}}$$

$$MR = \frac{M_{SN}}{M_{NS}} = \frac{\dot{m}_{SN}V_{SN}^2}{\dot{m}_{NS}V_{NS}^2} = \frac{\rho_{SN}Q_{SN}V_{SN}^2}{\rho_{NS}Q_{NS}V_{NS}^2} = \frac{\rho_{SN}A_{SN}V_{SN}^3}{\rho_{NS}A_{NS}V_{NS}^3} = \frac{\rho_{SN}A_{NS}^2Q_{SN}^3}{\rho_{NS}A_{SN}^2Q_{NS}^3}$$

For the prototype model we calculate that the $\frac{Q_{SN}}{Q_{NS}} = 5, \frac{A_{NS}}{A_{SN}} = 1, \rho_S = 1.1$ and $\rho_N = 1.2$ and for the model we have $\frac{A_{SN}}{A_{NS}} = \frac{0.75}{0.88}$ Therefore;

$$(MR)_{model} = (MR)_{Prototype}$$

$$(\frac{\rho_{SN}A_{SN}V_{SN}^3}{\rho_{NS}A_{NS}V_{NS}^3})_m = (\frac{\rho_{SN}Q_{SN}V_{SN}^2}{\rho_{NS}Q_{NS}V_{NS}^2})_p$$

$$(\frac{1}{1.075} \times \frac{0.75}{0.88} \times \frac{V_{SN}^3}{V_{NS}^3})_m = (\frac{1.1}{1.2} \times 1 \times 5^3)_p$$

$$(\frac{V_{SN}}{V_{NS}})_{mo} = 5.084$$

So, keep the $V_{SN} = 1.0, so\ V_{NS}$ will be equal to 0.2.

Parameter	Nondimensionalization
Inlet velocity for upper layer	$\widetilde{U}_{in,1} = 1$
Inlet velocity for lower layer	$\widetilde{U}_{in,2} = 0.5$
Channel Length	$\tilde{L} = 20$
Channel Height	$\widetilde{H} = 1$
Salinity for upper layer	$\tilde{S}_S = 0$
Salinity for lower layer	$\tilde{S}_N = 1$

Table S1.2: Nondimensional Parameters for the Simulation Setup

S1.5: Boundary Conditions and Numerical Method

The governing equations are solved using the high-order spectral element method (SEM) incompressible Navier-Stokes solver Nek5000 [7]. Nek5000 is an open-source solver, designed for solving the incompressible Navier–Stokes equations and transport equations (e.g., salinity, temperature, scalar tracers) for different boundary conditions. For velocity the boundary conditions are, wall (no-slip) at the bottom and symmetry (slip) at the top of the domain. The inlets are and outlets are marked in Figure 1, and all the inlets are defined as homogeneous Dirichlet that is constant inflow, and all the outlets are defined as Neumann that is free-outflow. The little bump between inflow and outflow is defined as wall. For salt, top and bottom wall are defined as no-flux boundaries that is they do not have any effect on the concentration of the salt. For salt, Inflows are homogeneous Dirichlet and outflows are Neumann. For initial condition, the flow has zero velocity inside the domain, with left side of the domain being filled with higher density (salinity) fluid corresponding to North side of the lake and the right side of the domain being filled with lower density (salinity) fluid corresponding to the South side of the lake. A lock-exchange is initiated at the center of the domain and the flow is allowed to develop over time.

In SEM, the flow domain is divided into small blocks called elements. Inside each element, the solution is approximated with high-order Lagrange polynomials going through Gauss–Lobatto–Legendre (GLL) point distribution (see Fig. S1.2). The Pn-Pn scheme is used for discretizing the velocity and pressure, which means across elements both velocity and pressure are represented by Lagrange polynomial of the same order [12]. The non-linear advection term is computed with a polynomial 3/2 times that used for the other operators. This is done in order to avoid de-aliasing error. Nek uses a 3rd order accurate semi-implicit scheme, which is a combination of 3rd order Explicit (EXT) and 3rd order Backward difference (BDF). The advection and body-force terms are integrated explicitly in time, whereas the diffusion term is computed implicitly and is solved using an iterative Helmholtz solve. The pressure term is computed iteratively by solving the Pressure-Poisson equation, which obtained when the pressure term computed implicitly [11].

Nek5000 in the past has been successfully used to simulate and study many different buoyancy-driven flows, from saline-gravity currents [5] to buoyant plumes and jets [8-10]. The simulations were run on the University of Utah Center for High Performance Computing (CHPC). Three cases with different berm heights (0.16, 0.3, and 0.6), and the corresponding nondimensional input parameters are summarized in Table S1.3.

Berm Height	Re	$\mathrm{Fr_d}$	V_{SN}	V_{NS}	Number of computational points
0.16	10,000	0.506	1	0.5	1.44 million
0.3	10,000	0.506	1	0.2	1.44 million
0.6	10.000	0.506	1	0.2	1.44 million

Table S1.3. Summary of input parameters for three simulation cases with different berm heights.

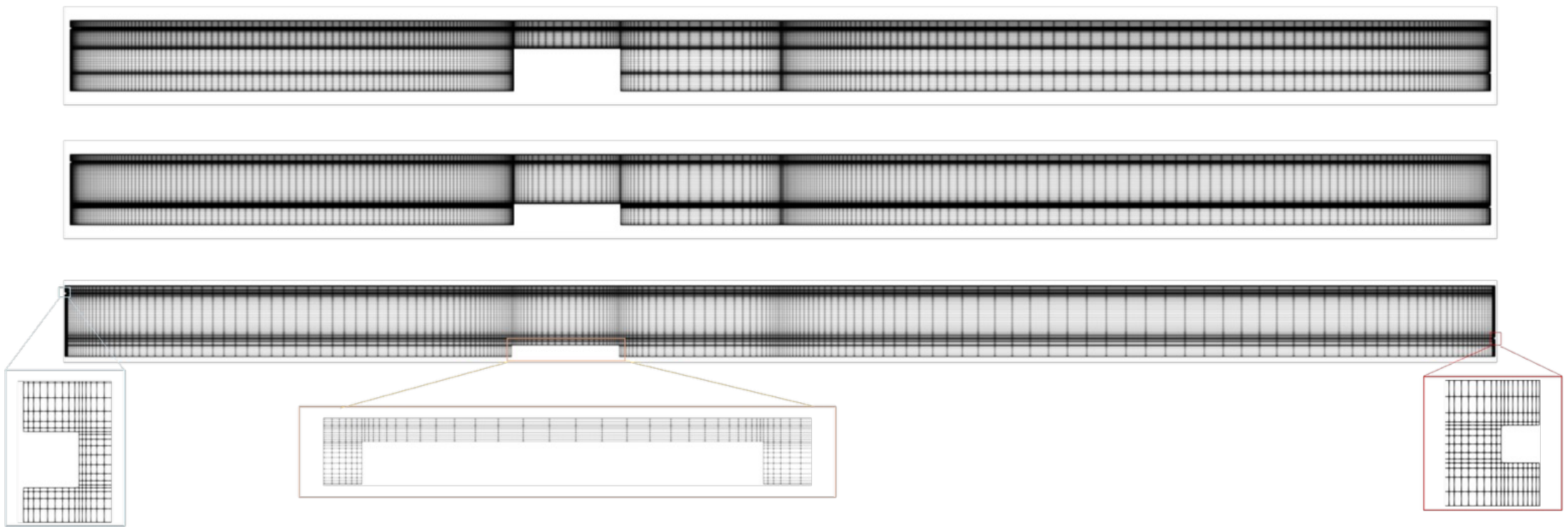

Figure S1.2: Mesh configurations corresponding to the three berm height cases summarized in Table S1.3.

The simulations employed a polynomial order of 7, with 29,321 spectral elements, resulting in about 1.4 million computational points. For the berm height of the 0.16, the simulation was carried out on 128 CPUs on Utah CHPC, requiring approximately 20 hours of wall-clock time to complete, whereas the 0.30 and 0.60 berm cases were executed on a single CHPC node (64 CPU cores) and required approximately two days of wall-clock time. The simulations were advanced in time with a nondimensional timestep of $\Delta t = 10^{-5}$ for the 0.16 berm height case. Grid independence analysis was conducted by running the 0.16 berm simulation with about 0.72 and 2.88 million computational points, and no qualitative difference was observed across the cases.

Particles are added to the flow to visualize the transport of microbes. The particles are Lagrangian tracers, which are neutrally buoyant particles that are small enough to have negligible Stokes number, which means they exactly follow the flow. So the equation that represents their motion are $dx_p/dt = v_x$ and $dy_p/dt = v_y$ where x_p and y_p are the coordinates of the particle and v_x and v_y are velocity of the particle. Particle velocities are obtained by interpolating the fluid velocity to the particle location [13]. The particles are labelled based on the flow origin, that is particles starting in the NS flow is labelled differently from particles starting in SN flow. During visualization, they colored according to their origin label.

References

[1] Rasmussen, M., Dutta, S., Neilson, B. T., & Crookston, B. M. (2021). CFD model of the density-driven bidirectional flows through the West Crack Breach in the Great Salt Lake Causeway. *Water*, *13*(17), 2423.

[2] Deville, M. O., Fischer, P. F., & Mund, E. H. (2002). *High-order methods for incompressible fluid flow* (Vol. 9). Cambridge university press.

[3] Fritts, D. C., Palmer, T. L., Andreassen, Ø., & Lie, I. (1996). Evolution and breakdown of Kelvin–Helmholtz billows in stratified compressible flows. Part I: Comparison of two-and three-dimensional flows. *Journal of Atmospheric Sciences*, *53*(22), 3173-3191.

[4] Zhou, Y. (2024). Hydrodynamic Instabilities and Turbulence: Rayleigh–Taylor, Richtmyer–Meshkov, and Kelvin–Helmholtz Mixing. Cambridge University Press.

[5] Marshall, C. R., Dorrell, R. M., Dutta, S., Keevil, G. M., Peakall, J., & Tobias, S. M. (2021). The effect of Schmidt number on gravity current flows: The formation of large-scale three-dimensional structures. Physics of Fluids, 33(10).

[6] Dunn, D., Crookston, B. M., Phillips, C., Dutta, S., & Neilson, B. (2025). Seasonal water and salt cycling in the Great Salt Lake after opening the new causeway breach. Journal of Hydrology: Regional Studies, 59, 102332.

[7] Fischer, P. F., Lottes, J. W., & Kerkemeier, S. G. (2008). nek5000 Web page.

[8] Özgökmen, T. M., Fischer, P. F., Duan, J., & Iliescu, T. (2004). Three-dimensional turbulent bottom density currents from a high-order nonhydrostatic spectral element model. Journal of Physical Oceanography, 34(9), 2006-2026.

[9] Fabregat, A., Gisbert, F., Vernet, A., Dutta, S., Mittal, K., & Pallarès, J. (2021). Direct numerical simulation of the turbulent flow generated during a violent expiratory event. Physics of Fluids, 33(3).

[10] Fabregat Tomàs, A., Poje, A. C., Özgökmen, T. M., & Dewar, W. K. (2016). Dynamics of multiphase turbulent plumes with hybrid buoyancy sources in stratified environments. Physics of Fluids, 28(9).

[11] Deville, M. O., Fischer, P. F., & Mund, E. H. (2002). High-order methods for incompressible fluid flow (Vol. 9). Cambridge university press.

[12] Mittal, K., Dutta, S., & Fischer, P. (2019). Nonconforming Schwarz-spectral element methods for incompressible flow. Computers & Fluids, 191, 104237.

[13] Mittal, K., Parik, A., Dutta, S., Fischer, P., Kolev, T., & Lottes, J. (2025). General field evaluation in high-order meshes on GPUs. Computers & Fluids, 295, 106606.

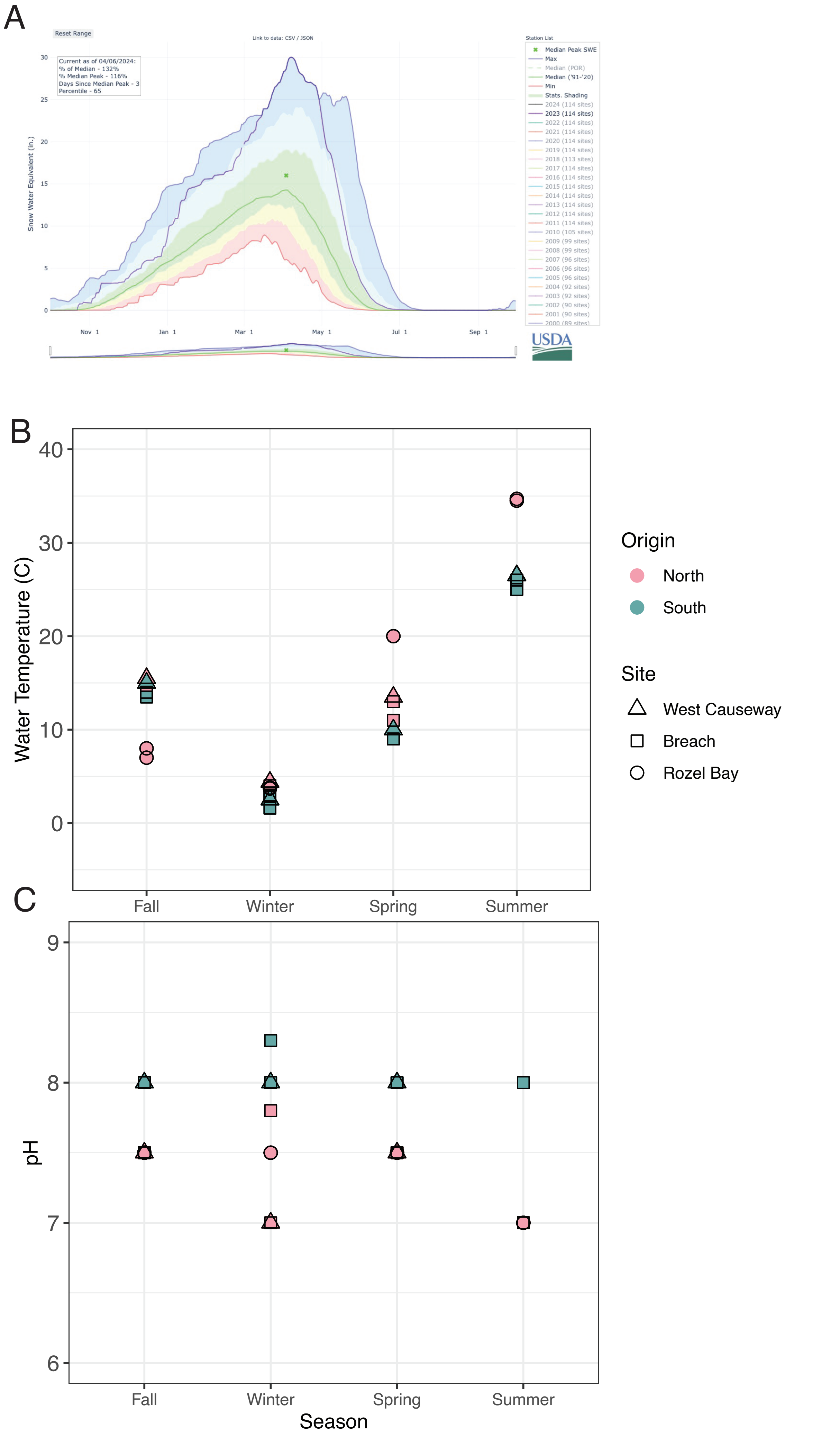

A
Reset Range
Link to data: CSV / JSON
Current as of 04/06/2024:
% of Median - 132%
% Median Peak - 116%
Days Since Median Peak - 3
Percentile - 65
Snow Water Equivalent (in.)
30
25
20
15
10
5
0
Nov 1
Jan 1
Mar 1
May 1
Jul 1
Sep 1
Station List
Median Peak SWE
Max
Median (POR)
Median ('91-'20)
Min
Stats. Shading
2024 (114 sites)
2023 (114 sites)
2022 (114 sites)
2021 (114 sites)
2020 (114 sites)
2019 (114 sites)
2018 (113 sites)
2017 (114 sites)
2016 (114 sites)
2015 (114 sites)
2014 (114 sites)
2013 (114 sites)
2012 (114 sites)
2011 (114 sites)
2010 (105 sites)
2009 (99 sites)
2008 (99 sites)
2007 (96 sites)
2006 (96 sites)
2005 (96 sites)
2004 (92 sites)
2003 (92 sites)
2002 (90 sites)
2001 (90 sites)
USDA
B
Water Temperature (C)
40
30
20
10
0
Fall
Winter
Spring
Summer
Origin
North
South
Site
West Causeway
Breach
Rozel Bay
C
pH
9
8
7
6
Fall
Winter
Spring
Summer
Season

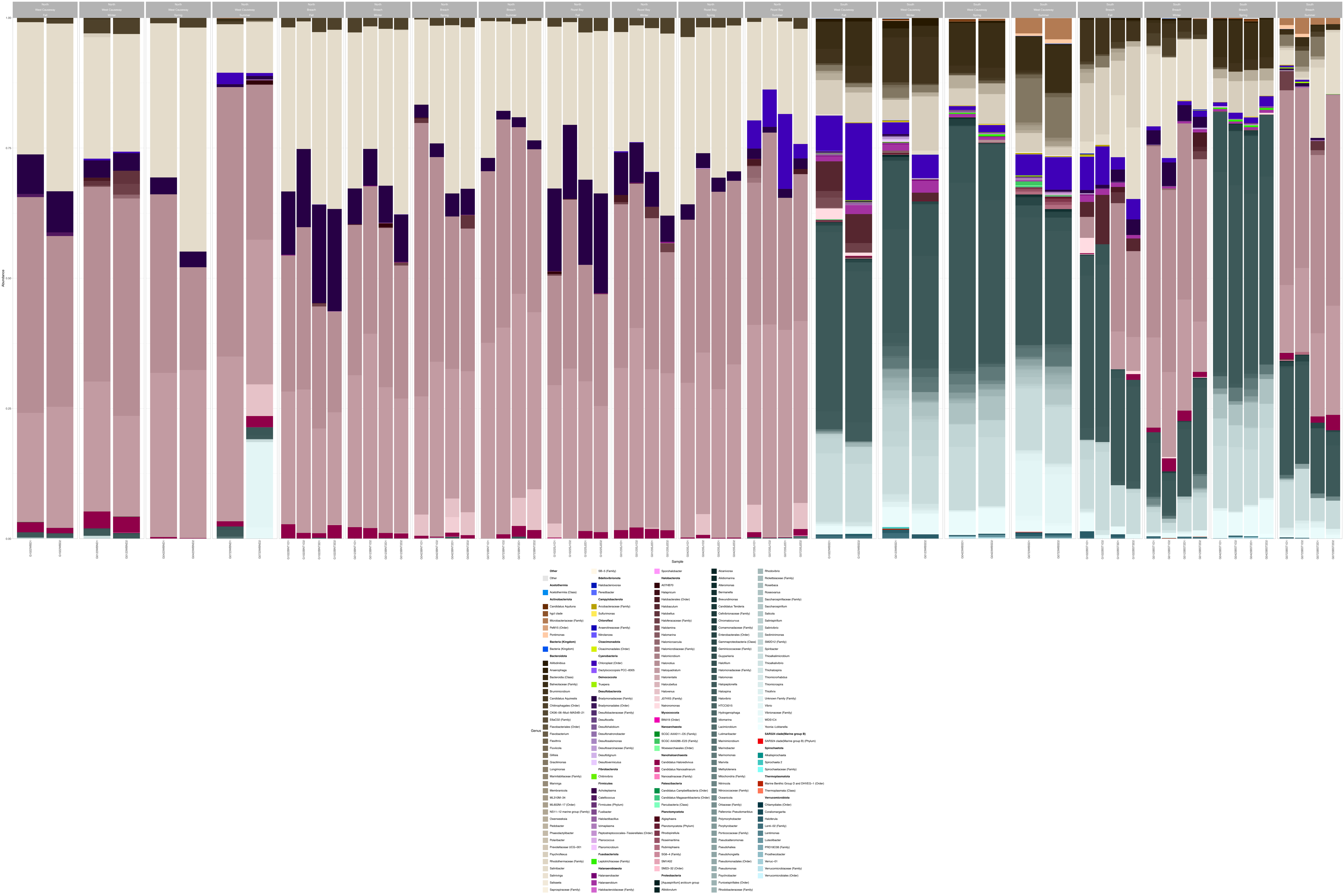
Abundance
Sample
Genus
